# Distribution of three congeneric shrub species along an aridity gradient is related to seed germination and seedling emergence

**DOI:** 10.1093/aobpla/plv071

**Published:** 2015-07-02

**Authors:** Liming Lai, Yuan Tian, Yongji Wang, Xuechun Zhao, Lianhe Jiang, Jerry M. Baskin, Carol C. Baskin, Yuanrun Zheng

**Affiliations:** 1 Key Laboratory of Resource Plants, Beijing Botanical Garden, West China Subalpine Botanical Garden, Institute of Botany, Chinese Academy of Sciences, Xiangshan, Beijing, China; 2 Fukang Station of Desert Ecology, Xinjiang Institute of Ecology and Geography, Chinese Academy of Sciences, South Beijing Road, Urumqi, Xinjiang, China; 3 Department of Biology, University of Kentucky, Lexington, KY 40506, USA; 4 Department of Plant and Soil Sciences, University of Kentucky, Lexington, KY 40546, USA

**Keywords:** *Caragana*, geographical distribution, precipitation gradient, sand burial, seed germination, seedling emergence

## Abstract

Environmental tolerance of a species has been shown to correlate positively with its geographical range. On the Ordos Plateau, three *Caragana* species are distributed sequentially along the precipitation gradient. We hypothesized that this geographical distribution pattern is related to environmental tolerances of the three *Caragana* species during seed germination and seedling emergence stages. To test this hypothesis, we examined seed germination under different temperature, light and water potentials, and monitored seedling emergence for seeds buried at eight sand depths and given different amounts of water. Seeds of *C. korshinskii* germinated to high percentages at 5 : 15 to 25 : 35 °C in both light and darkness, while those of *C. intermedia* and *C. microphylla* did so only at 15 : 25 and 25 : 35 °C, respectively. Nearly 30 % of the *C. korshinskii* seeds germinated at −1.4 MPa at 20 and 25 °C, while no seeds of the other two species did so. Under the same treatments, seedling emergence percentages of *C. korshinskii* were higher than those of the other two species. The rank order of tolerance to drought and sand burial of the three species is *C. korshinskii* > *C. intermedia* > *C. microphylla*. The amount of precipitation and sand burial depth appear to be the main selective forces responsible for the geographical distribution of these species.

## Abstract (precis)

In this study we aimed to determine whether a sequential distribution pattern along an aridity gradient is related to seed germination and seedling emergence of three *Caragana* species. The study tested the adaptive abilities of these species to major sandy environment factors including soil water potential, precipitation amount, and sand burial depth. The rank order of tolerance to drought and sand burial of the three species is *C. korshinskii*>*C. intermedia*>*C. microphylla*. The amount of precipitation and sand burial depth appear to be the main selective forces responsible for the geographical distribution of these species.

## Introduction

The growth and distribution of a plant is limited if any environmental factor exceeds its tolerance (Gates *et al*. 1956). Environmental tolerance has been shown to correlate positively with the geographical range of a species (Brown 1984; Slatyer *et al*. 2013). Species with broad environmental tolerances can occupy a wide variety of habitats and thus achieve an extensive geographical distribution (Gaston and Spicer 2001). Thus studies of the environmental tolerances of plants, especially during life history stages of germination and seedling establishment, can contribute to our understanding of the relationship between their distribution and environmental factors (Gaston and Spicer 2001; Brändle *et al*. 2003; Slatyer *et al*. 2013; Wasof *et al*. 2013).

The environmental control of seed germination is often a complex process, and seeds can only germinate when environmental factor stresses do not exceed their tolerances (Baskin and Baskin 2014). Temperature, light and water are the most frequently discussed factors that regulate germination (Chauhan and Johnson 2008). Temperature requirements for germination vary across species (Baskin and Baskin 2014). Seed responses to light can control germination time and influence the survival of seedlings (Zheng *et al*. 2005*a*). Differing water availability requirements for germination in different species can partly reflect their adaptation to drought, which is related to habitat requirements (Baskin and Baskin 2014).

After a seed germinates below ground, the seedling must elongate in order to emerge at the soil surface and survive. In sandy areas, seeds are buried at different depths. Burial can be beneficial by providing a moist environment and by preventing damage from herbivores, or it can be detrimental by preventing seedling emergence (Maun 1998; Zheng *et al*. 2005*c*). In fact, sand burial is a major factor controlling the distribution and composition of vegetation in a desert ecosystem (Maun 1998). Plant species distributed in arid and semi-arid areas, where precipitation is unpredictable and the amount is low, have special drought tolerance requirements during seedling emergence (Zhu *et al*. 2014). Thus, the geographical distribution of a species may correspond to its ability to tolerate water stress and sand burial at the seedling emergence stage of the life cycle.

The papilionoid legume species *Caragana korshinskii*, *C. intermedia* and *C. microphylla* are three of the dominant perennial shrub species on the Ordos Plateau. *Caragana korshinskii*, *C. intermedia* and *C. microphylla* are distributed primarily in the western, middle and eastern parts of the Ordos Plateau, respectively (Zhao 1991). The reason(s) for this phenomenon is (are) unclear. Although there have been many studies on species distribution in relation to environmental gradients, most of them have focused on comparing plant species at two distant regions (Wasof *et al*. 2013).

We hypothesized that the geographical distribution of three *Caragana* species along the aridity gradient are broadly related to their environmental tolerances during germination and seedling establishment. To test this hypothesis, we sought answers to the following questions: (i) Do the three *Caragana* species differ in environmental tolerances during seed germination and seedling emergence stages under key environmental factors, including temperature, light, water and sand burial, and if so, how? (ii) If there are differences in environmental tolerances during these two life history stages, how are they related to the east to west precipitation and sand burial depth gradients?

## Methods

### Study sites

The Ordos Plateau is located in the southern part of Inner Mongolia in northern China. The climate is continental, with an extreme variation in seasonal and diurnal temperatures. Mean monthly temperatures are <5 °C from November to March, and they range between 7.4 and 21.9 °C from April to October. Annual precipitation decreases gradually from 400 mm in the east to 150 mm in the west, and ∼93 % of the annual precipitation occurs from April to October (Zheng *et al*. 2006). Further, vegetation cover decreases from east to west and frequency of sand burial by wind increases (Xu 2006). Elevation ranges from 850 to 1600 m above sea level. The general landscape of the Plateau consists mainly of lowland meadows and fixed, semi-fixed and moving sand dunes. Soils on the Ordos Plateau are brown pedocals, castanozems and dark loessial soils (Zheng *et al*. 2006); soils in the sandy lands belong to the first two soil types.

### Seed collection and storage

Seeds of *C. korshinskii*, *C. intermedia* and *C. microphylla* were collected from east to west along the precipitation gradient across the Ordos Plateau in September, 2005. Seeds were collected randomly from many plants to get an adequate representation of genetic diversity. After the seeds were cleaned and air dried, they were stored in cloth packets at 4 °C until used.

### Germination experiments

Germination experiments were carried out in temperature-, light- and humidity-controlled growth chambers, in 2007 and 2009. The chambers were equipped with cool white fluorescent lights and set for a daily photoperiod of 14 : 10 h (day : night).

The seeds were surface sterilized by soaking them in a 0.52 % sodium hypochlorite solution for 1 min and were then rinsed several times with distilled water (Zheng *et al*. 2005*a*). Then, they were placed in 90 mm diameter × 15 mm deep Petri dishes on three layers of filter paper. Distilled water was added until the seeds floated but were not inundated. Each treatment had 5 replicates of 25 seeds. Treatments were inspected daily under a dim fluorescent light (10 µmol m^−2^ s^−1^). Emergence of the radicle was taken as the criterion for germination (Baskin and Baskin 2014), and they were discarded after counting. Tests were terminated after 30 days (Zheng *et al*. 2005*a*).

#### Effects of temperature and photon irradiance on germination

The effects of fluctuating temperature regimes and of photon irradiance (400–700 nm) on germination were tested at 5 : 15, 10 : 20, 15 : 25, 20 : 30 and 25 : 35 °C (night : day). These five temperature regimes are close to those that occur in spring and summer in semi-arid areas in the study area (Qi 1998). Light levels were 125, 75, 25 and 0 (dark) µmol m^−2^ s^−1^ at each of the five temperature regimes. The seeds were incubated in clear plastic boxes covered with layers of white and black plastic netting to achieve the different light levels or in black wooden boxes made with two layers spaced 4 cm apart. In each layer, there was a long, narrow aperture, and the aperture in one layer was offset from that in the other layer. This construction allowed air to flow freely into and out of the box but prevented light from entering it.

#### Effects of temperature and water potential on germination

Different water potentials (WPs) were obtained using solutions of polyethylene glycol 6000 (PEG-6000) following the method of Michel and Kaufmann (1973). Water potentials were 0, −0.2, −0.4, −0.6, −0.8, −1.0, −1.2, −1.4, −1.6, −1.8 and −2.0 MPa. The experiments were carried out in darkness at 10, 15, 20, 25 and 30 °C.

### Seedling emergence experiment

#### Effects of sand burial and water supply regime on seedling emergence

This experiment tested the effects of sand burial and water supply regime on seedling emergence at 15 : 25 °C (night : day) and a photon irradiance of 125 µmol m^−2^ s^−1^. Sand was collected from the natural habitats of each of the three species and sifted to remove debris. It was then heated in an oven at 100 °C for 48 h to kill any seeds that might affect the results. Plastic pots (6.8 cm diameter and 16 cm height) were used in the experiment. Holes at the bottoms of the pots were covered with nylon-net to prevent loss of sand but allow drainage of excess water. The pots were filled with sand to the given depths for seed placement, and then seeds were sown on the sand surface and additional sand was added to the pots.

There were eight sand burial depths: 0 (surface), 0.5, 1, 1.5, 2, 3, 4 and 6 cm. The pots were watered only once during the entire period of the experiment, at the beginning with distilled water. There were eight water regimes: 2.5, 5, 7.5, 10, 15, 20, 30 and 40 mm. Each treatment had five replicates of 25 seeds. The number of seedlings that emerged above the sand surface was recorded daily. Tests were terminated after 30 days.

### Statistical analysis

Germination was measured using two indices: final germination percentage (FGP) and germination rate (GR). Final germination percentage is the percentage of seeds sown that actually germinated. Germination rate (i.e. speed) was estimated using a modified Rozema index of GR (Rozema 1975): $\sum 100(G_{i}/nt_{i}),$ where *n* is the number of seeds used in a treatment (25 each replicate) and *G_i_* is the number of seeds germinated on day *t_i_* (*t_i_* = 0, 1, 2, 3…). Higher values indicate faster germination. Final emergence percentage (FEP) and emergence rate (ER) were calculated in the same way as FGP and GR.

Two-way analysis of variance (ANOVA) was used to analyse effects of temperature, photon irradiance and their interactions on FGP and GR; effects of temperature, WP and their interactions on FGP and GR; and effects of burial depth, water supply and their interactions on FEP and ER. Tukey's test was used to determine differences of FGP and GR among different treatments for the same species, including: (i) at the same temperature under different light treatments and (ii) under the same light treatment at different temperatures. The relationship between FEP and water supply regime was tested with nonlinear regression analyses. All statistical analyses were performed using the SPSS 13.0 package (SPSS 2004).

## Results

### Effects of temperature and photon irradiance on germination

**Figure 1.**
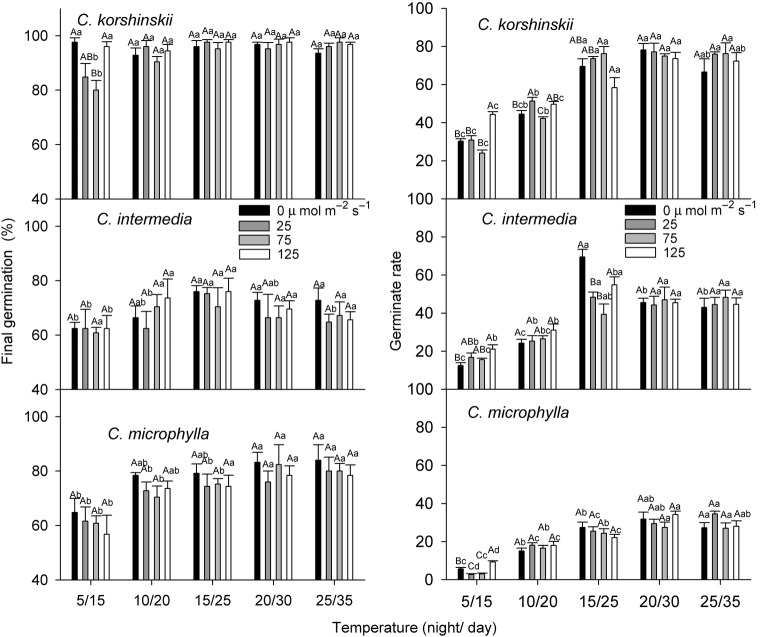
Effect of temperature and photon irradiance on FGP and GR (mean ± SE, *n* = 5) of three *Caragana* species. Different uppercase letters indicate significant differences at the same temperature under different light treatments, while different lowercase letters indicate significant differences under the same light treatment at different temperatures for the same species. *P* < 0.05.

The effects of temperature, photon irradiance and their interactions were significant for FGP of all three species (Table 1). Seeds of *C. korshinskii* germinated well at all temperature regimes; FGPs ranged from 80.0 to 97.6. In contrast, seeds of the other two species had lower FGPs in the same treatments. Final germination percentages of *C. intermedia* seeds were highest at 15 : 25 °C. For *C. microphylla*, low temperature significantly inhibited germination, and FGP was lowest at 5 : 15 °C. Irradiance did not affect FGP in most treatments (Fig. 1).

**Table 1.** Two-way ANOVA of FGP, GR, FEP, ER and their interaction of three *Caragana* species in relation to different environmental factors. df, numerator and denominator degrees of freedom; ns, not significant: *P* > 0.05; *P < 0.05; **P < 0.01.

Source	df	*C. korshinskii*		*C. intermedia*		*C. microphylla*	
		FGP	GR	FGP	GR	FGP	GR
Seed germination							
Temperature (T)	4, 80	7.5**	127.9**	4.4**	74.0**	48.3**	99.6**
Light intensity (L)	3, 80	3.9*	1.3ns	3.4*	1.8ns	3.7**	1.6ns
T × L	12, 80	3.4**	3.6**	1.9*	3.4**	3.4**	1.7ns
Seed germination							
Temperature (T)	4, 220	303.0**	466.2**	28.0**	90.4**	73.9**	179.7**
Water potential (WP)	10, 220	408.0**	642.5**	215.0**	206.1**	475.0**	442.3**
T × WP	40, 220	13.5**	28.2**	4.3**	13.2**	8.4**	24.2**
Seedling emergence		FEP	ER	FEP	ER	FEP	ER
Burial depth (D)	7, 256	377.2**	235.7**	221.8**	169.5**	535.3**	268.7**
Water supply (W)	7, 256	624.3**	504.1**	165.9**	189.2**	231.0**	186.6**
D × W	49, 256	38.1**	23.3**	15.2**	14.7**	28.0**	17.9**

Temperature, but not irradiance, had a significant effect on GR (two-way ANOVA, Table 1). Germination rate of the three *Caragana* species was increased by higher temperature, and seeds germinated fastest at 15 : 25 or 20 : 30 °C. At low temperature (5 : 15 °C), light increased GR, but no stimulating effects were found at higher temperatures (Fig. 1).

### Effects of temperature and water potential on germination

**Figure 2.**
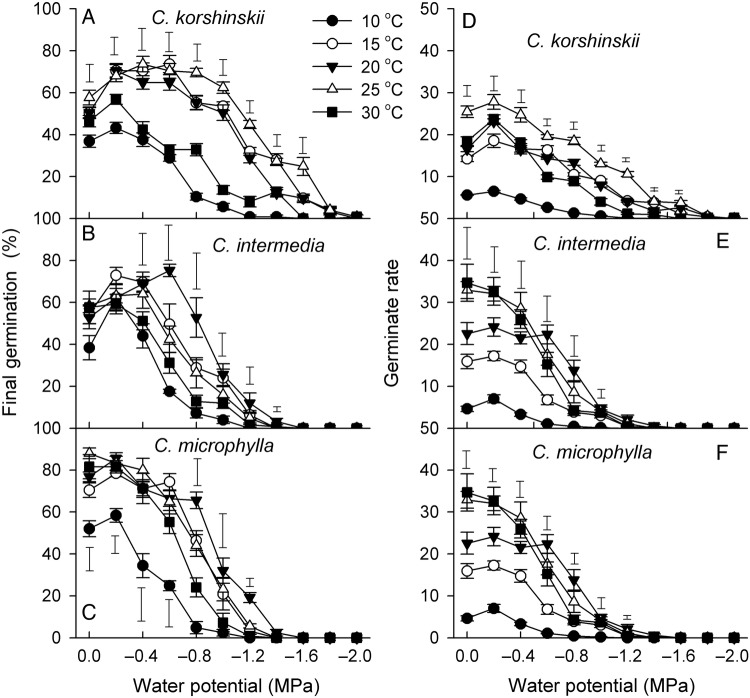
Final germination percentages and rates (mean ± SE, *n* = 5) of three *Caragana* species under different temperatures and WPs. Error bars above (below) the data points are the LSD values at *P* = 0.05, and no error bar means not significant (*P* > 0.05).

Temperature, WP and their interactions significantly affected FGP and GR in all three species (Table 1). At the same WP, FGP was significantly higher at 15, 20 and 25 °C than at the other two temperatures, while GR decreased with temperature (*P* < 0.05, Fig. 2). Final germination percentage increased as WP decreased from 0 to −0.2 MPa at all five temperatures (except for *C. microphylla* seeds at 25 °C). The WP at which FGP began to decrease and at which it approached 0 % varied among species and temperatures. A WP of −1.0 MPa had a less negative effect on germination percentages of *C. korshinskii* than on the other two species. At 20 and 25 °C, nearly 30 % of the *C. korshinskii* seeds germinated at −1.4 MPa, while no seeds of *C. intermedia* or *C. microphylla* germinated at this WP (Fig. 2).

### Effects of sand burial and water supply regime on seedling emergence

**Figure 3.**
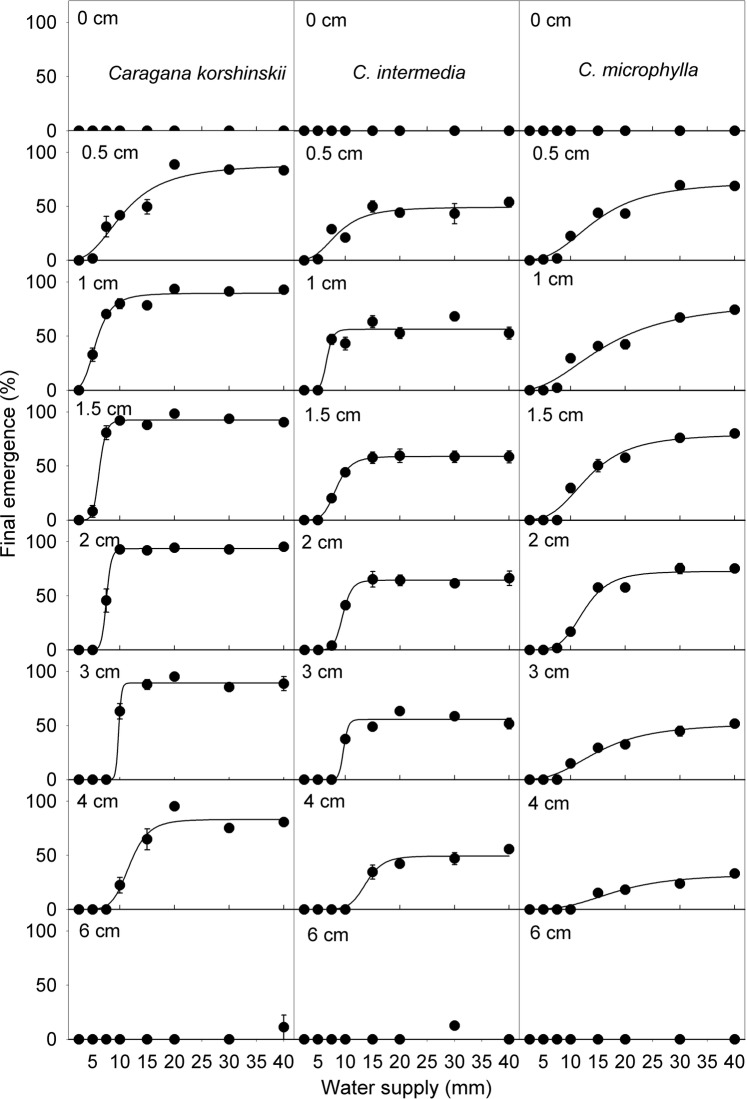
Final emergence percentage (mean ± SE, *n* = 5) of three *Caragana* species under different sand burial depths and watering regimes at 15 : 25 °C. Other abbreviations are the same as in Fig. 2.

Burial depth, water supply regime and their interactions had significant effects on both FEP and ER for all three species (Table 1). In general, seeds of neither species germinated on the sand surface. Further, only a very few seedlings emerged from a burial depth of 6 cm, regardless of water supply regime (Fig. 3), and no seedlings emerged from any depth in the 2.5 mm water supply treatment. Final emergence percentage was highest under sand burial depths of 0.5–3 cm. With increased burial depth, FEP decreased, and a higher amount of water was needed for seedling emergence. The sigmoidal curve was used to fit the relationship between water supply and FEP [*R*^2^ ranged from 0.94 to 0.99 for *C. korshinskii*, df (2, 5), *P* < 0.001; from 0.90 to 0.99 for *C. intermedia*, df (2, 5), *P* < 0.05; from 0.95 to 0.97 for *C. microphylla*, df (2, 5), *P* < 0.001]. The curves showed that FEP would reach the maximum under proper water supply (Fig. 3).

The highest FEP for *C. korshinskii* occurred under the 20 mm water supply regime at burial depths of 0.5–4 cm. Under water supply regimes of 7.5–15 mm, seedling emergence of all three species increased as burial depth increased from 0.5 to 2 cm, but then it decreased at burial depths >2 cm. With 5 mm water supply, seedlings of all three species emerged at shallow burial depths (0.5–1.5 cm).

Similar to *C. korshinskii*, FEP of *C. intermedia* increased as sand burial depth increased from 0.5 to 2 cm. This range of burial depths was also optimal for seedling emergence of *C. intermedia*. Final emergence percentage decreased significantly at burial depths >2 cm. At least 15 mm of water was required for seedling emergence from 4 cm.

For *C. microphylla*, there was a positive correlation between FEP and water supply regime at the same burial depth. The highest FEP was at 1.5 cm burial depth with 40 mm water supply. Under the same water supply regime at burial depths of 3–4 cm, FEP was significantly lower than it was for the other two species. Burial depths of 0.5–1.5 cm with water supply regimes of 30–40 mm were the optimal conditions for seedling emergence.

**Figure 4.**
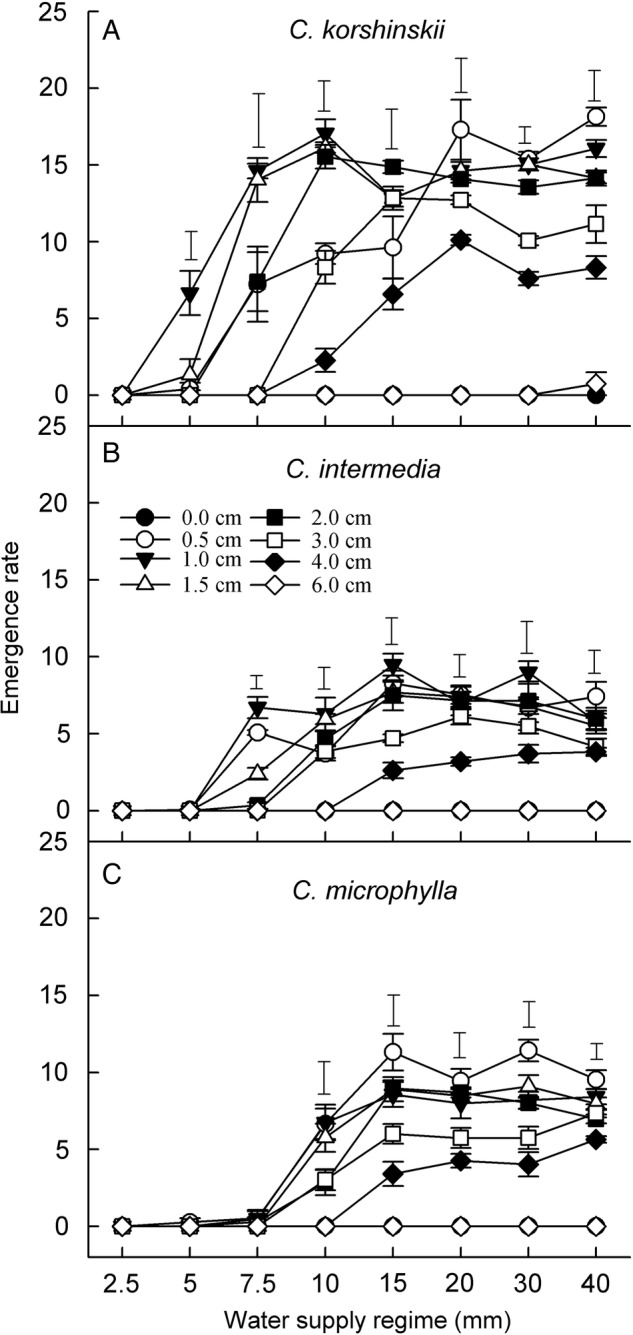
Emergence rate (mean ± SE, *n* = 5) of three *Caragana* species under different sand burial depth and water supply regimes at 15 : 25 °C. Other abbreviations are the same as in Figure 2.

Seedling ERs of all three species were negatively correlated with burial depth (Fig. 4). Emergence rate of *C. korshinskii* increased with water supply, until the maximum was reached at 20 mm. At burial depths of 0.5–2 cm, ER was significantly higher than it was at 3 and 4 cm. Emergence rate for both *C. intermedia* and *C. microphylla* was lower than that of *C. korshinskii* under the same treatment. At burial depths ≥3 cm, ER of all three species significantly decreased.

## Discussion

### Responses of seed germination to temperature, light and water potential

When the water supply is adequate, temperature and light are the most important abiotic factors controlling seed germination (Baskin and Baskin 2014). Seeds of each of the *Caragana* species germinated equally well under the three photon irradiances and in darkness across the range of temperatures tested, but germination percentages were higher in *C. korshinskii* than in *C. intermedia* and *C. microphylla* (Fig. 1). Thus, the light/dark requirements for germination would not play a role in habitat selection of these three species. Wang *et al*. (2009) reported that nondormant seeds of *C. arborescens* and *C. roborovskyi* germinated equally well in light and darkness.

Precipitation in deserts varies greatly in both amount and timing. Many plant species have mechanisms that regulate germination occurring under favourable conditions (Adondakis and Venable 2004). Germination of the three *Caragana* species in our study differed in responses to WP. Seed germination of all three *Caragana* species was strongly inhibited by WPs lower than −1.0 MPa, which is similar to the inhibition found for some other desert species (Tobe *et al*. 2005; Zheng *et al*. 2005*b*). Seeds of *C. korshinskii* germinated to higher percentages at WPs of −1.0 to −1.6 MPa than the other two species (*P* < 0.05). This indicated the higher drought tolerance of *C. korshinskii* seeds. In general the GR for seeds of *C. korshinskii* was lower (the seeds required more days to germinate) than that of the other two species (Fig. 1). Our field observation revealed that long intermittent drought periods are usual, which are fatal to the fast germinators.

### Relationships of seedling emergence traits and distribution

Generally, germination is inhibited for seeds sown on the surface due to excessive temperature fluctuations, desiccation and especially evaporative stress (Zhang and Maun 1990). In our study, hardly any seeds sown on the surface germinated, but they could germinate to high percentages when transferred to wet filter paper in Petri dishes. Thus, lack of germination on the surface was due to environmental stress and not to loss of viability.

In the natural environment, seeds can be buried to varying depths depending on prevailing wind velocities and habitat characteristics (Maun 1998). In the three *Caragana* species, shallow burial and the appropriate water supply amount stimulated germination. However, seedling emergence percentages were decreased by relatively deep burial depth, and very few seedlings emerged from a depth of 6 cm (Fig. 3). There are several possible reasons for this. First, mass of the sand above the seed is a physical obstacle that may be difficult to overcome by an elongating seedling. Second, seeds at deeper depths may acquire dormancy due to higher soil moisture, lower temperatures, poor gas exchange and especially oxygen deficiency (Baskin and Baskin 2014; Maun 1998; Tobe *et al*. 2005). However, it is unlikely that seeds of legumes that are nondormant at maturity, and even those that have physical dormancy at maturity and then become nondormant, can be induced into dormancy (Baskin and Baskin 2014).

In the sandy environment, blowing sand decreases with increase in vegetation cover (Xu 2006). On the Ordos Plateau, vegetation cover decreases from east to west (Zheng *et al*. 2006), and thus the possibility of sand burial at deeper depths likewise increases. Seedling emergence tolerance to deeper sand burial of the three species differs, and this is an important reason why *C. korshinskii*, *C. intermedia* and *C. microphylla* occur in the western, middle and eastern parts of the Ordos Plateau, respectively.

In arid and semi-arid areas, the annual amount of precipitation is the main limiting factor for plant establishment (Gutterman 1993). Seedling emergence responses to the water supply regimes differed among the three *Caragana* species in our study. In the field, a long interval usually occurs between two precipitation events. The ability of seeds and seedlings of the three species to utilize the small amount of precipitation for germination, emergence and survival is a determinant of their distribution.

### Distribution of three *Caragana* species: a conceptual model

**Figure 5.**
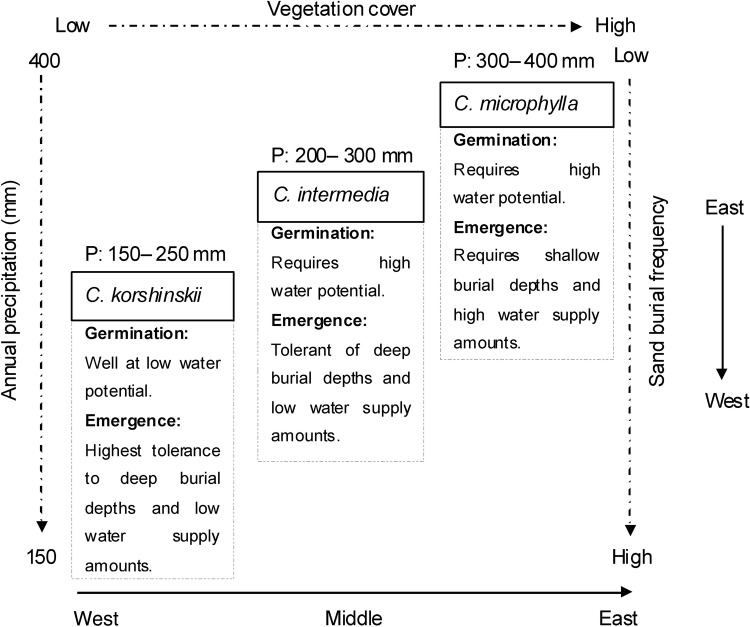
Conceptual model of the distribution of three *Caragana* species along the precipitation (P) gradient.

Differences in environmental tolerances to temperature, water supply and sand burial depth of seed germination and seedling emergence of the three *Caragana* species are correlated with the sequential east to west distribution of environmental factors on the Ordos Plateau (Fig. 5). The seed/seedling stage of *C. microphylla* is least tolerant to aridity and burial, which we suggest plays a role in restricting its distribution primarily to the eastern part of the Ordos Plateau. On the other hand, the seed/seedling stage of *C. korshinskii* is the most tolerant to aridity and is restricted to the western part of the Plateau. *C. intermedia*, with moderate tolerance to aridity and sand burial in the seed to seedling stage occurs in the middle of the gradient, between *C. microphylla* in the east and *C. korshinskii* in the west. Thus, precipitation amount and sand burial appear to be selective forces that play an important role in the sequential distribution of *C. korshinskii*, *C. intermedia* and *C. microphylla* along the decreasing west to east aridity gradient on the Ordos Plateau. The west to east increase in vegetation cover, decrease in sand burial and increase in amount of precipitation are interrelated with the distribution of the three *Caragana* species.

## Conclusions

In this study we examined the correlation of environmental tolerances of three *Caragana* species during their germination and seedling emergence stages and their sequential distribution pattern. Results showed that rank order of tolerance to drought and sand burial of the three species is *C. korshinskii* > *C. intermedia* > *C. microphylla*. In the sandy area, tolerance to drought and sand burial seem to be the main selective forces that play an important role in the sequential distribution pattern of these three species. The results can help with selection of plant species during restoration of vegetation.

## Sources of Funding

This work was funded by the National Natural Science Foundation of China (Grant No. 41330749 and 41401105).

## Contributions by the Authors

L.L. and Y.Z. conceived and designed the experiments. L.L., Y.T., Y.W. and X.Z. performed the experiments. L.L., L.J. and Y.Z. analysed the data. L.L., J.M.B., C.C.B. and Y.Z. wrote the manuscript.

## Conflict of Interest Statement

None declared.
